# Transformational Leadership and the Reporting of Patient Safety Events Among Emergency Nurses: The Mediating Role of Psychological Safety

**DOI:** 10.3390/healthcare14203396

**Published:** 2026-10-12

**Authors:** Nükhet Bayer, Ayşegül Turan, Seval Altunkeser

**Affiliations:** 1 Faculty of Nursing, Lokman Hekim University, 06510 Ankara, Turkey; 2 Department of Nursing, Faculty of Health Sciences, Kırşehir Ahi Evran University, 40100 Kırşehir, Turkey; aysegul.turan@ahievran.edu.tr; 3 Nursing Management Master’s Program, Graduate School of Health Sciences, Lokman Hekim University, 06510 Ankara, Turkey; sevalaltunkeser@gmail.com

**Keywords:** psychological safety, transformational leadership, patient safety event reporting, emergency departments, patient safety culture

## Abstract

**Highlights:**

**What are the main findings?**

• Psychological safety showed the strongest association with patient safety event reporting behavior among the variables examined in emergency department nurses.
• Transformational leadership was associated with reporting behavior both directly and indirectly through psychological safety, consistent with partial mediation.

**What are the implications of the main findings?**

• Developing nurse managers’ transformational leadership skills may foster nurses’ perceived psychological safety, which is associated with more frequent reporting.
• Strengthening patient safety event reporting may require attention to psychological safety, not formal reporting systems alone.

**Abstract:**

**Background/Objectives:** Underreporting of patient safety events is a persistent problem in high-risk clinical settings such as emergency departments, yet the managerial and psychological conditions associated with reporting remain underexamined. This study aimed to examine the relationship between transformational leadership and patient safety event reporting behavior among emergency department nurses, and to test the mediating role of psychological safety in this relationship. **Methods:** This descriptive, cross-sectional study was conducted with 295 nurses working in the emergency departments of two city hospitals in Ankara, Turkey. Data were collected using a personal information form, the Transformational Leadership Scale, the Psychological Safety Scale, and the “Reporting Patient Safety Events” composite of the Hospital Survey on Patient Safety Culture 2.0 (HSOPSC 2.0). Data were analyzed using descriptive statistics, confirmatory factor analysis, Pearson correlation analysis, and path analysis with bootstrap-based mediation testing (SPSS 29 and AMOS 24). **Results:** Transformational leadership was positively associated with psychological safety (β = 0.505). Psychological safety was positively associated with reporting behavior (β = 0.427). Transformational leadership was associated with reporting both directly (β = 0.167) and indirectly through psychological safety (β = 0.215, 95% CI [0.134, 0.305]), consistent with partial mediation. The model explained 28% of the variance in reporting behavior. **Conclusions:** The findings are consistent with psychological safety functioning as a partial mediator in the association between transformational leadership and patient safety event reporting. Strengthening nurse managers’ transformational leadership skills and fostering nurses’ perceived psychological safety may support reporting in emergency departments, although longitudinal and intervention studies are needed to confirm these relationships.

## 1. Introduction

Emergency departments are work environments that are structurally distinct from other units within a hospital. Unlike other units, emergency departments must cope with an unplanned and unpredictable patient demand [1]. The necessity of making high-risk decisions within a limited timeframe, combined with a shift-based work schedule, makes these units systematically more vulnerable to clinical errors [2]. Studies based on task analysis show that numerous potential error points have been identified in the workflow of emergency department nurses, and that the majority of these consist of action errors [3,4]. However, nursing errors occur in all hospital departments; the likelihood of errors increases in departments where the patient safety culture falls below the standard [2]. Under these conditions, it is unrealistic to expect error-free performance in the emergency department. In complex sociotechnical systems, what is feasible is not the complete prevention of errors, but rather the strengthening of organizational capacity to ensure that errors are detected, reported, and used as learning opportunities [5].

In the emergency department, the nurse’s behavior in reporting errors is just as critical as the identification of the error itself. However, various barriers stand in the way of this behavior. At the core of this lies the fact that reporting carries a real or perceived risk for the individual [6]. These barriers include fear of blame and disciplinary action, concern about damage to professional reputation, and a lack of confidence that reporting will lead to any change [7,8,9]. Reflecting these barriers, in a large-scale study, two-thirds of nurses reported that less than 20% of medication administration errors were reported [7]. This situation is a natural consequence of the management approach Reason [5] termed the “person-centered approach,” which views errors as moral failings and responds with discipline and blame. Indeed, in a patient safety culture study conducted at a university hospital in Turkey, the lowest scores were obtained in the dimensions of open communication about errors and managerial support; nurses assessed open discussion aimed at preventing errors and managers’ addressing safety concerns as the weakest areas [10]. However, hidden conditions within the system only become visible when someone reports them; therefore, a nurse’s reporting behavior directly determines the institution’s capacity to learn from errors.

Psychological safety is the shared belief that behaviors involving interpersonal risk—such as admitting mistakes—will not be punished within the work team [11]. From its inception, this concept has been used to explain differences in nurses’ behaviors regarding the recognition and reporting of errors [12]. In this study, psychological safety is addressed not as a shared climate at the team level, but rather as the level of safety perceived by individual nurses. Psychological safety is not a phenomenon that arises spontaneously within an organization. Theoretical and empirical work suggests that it is closely related to managers’ attitudes and behaviors, and transformational leadership theory offers a framework for understanding how managers may contribute to this sense of safety [13,14]. According to the theory, a manager, as a trustworthy role model, sets forth a security-focused vision (vision—inspiring others/serving as an appropriate role model). By framing error reporting not as a personal failure but as a contribution to a shared safety goal, the manager ensures that the team embraces this goal (acceptance of group goals). The manager legitimizes the open discussion of errors (intellectual encouragement). Through the individual relationships they build with each nurse, they convey the message that nurses will be supported rather than punished in the event of an error (individual attention). By defining high performance as a work environment where errors are identified and reported (high expectations for success), they make error reporting the norm [12]. This behavioral pattern is also reflected in the five-dimensional transformational leadership framework empirically derived by Podsakoff et al. [15]. Indeed, a systematic review conducted in the field of nursing has shown that transformational leadership is associated with increased error reporting and medication safety; in contrast, passive-avoidant and toxic leadership styles are associated with a decrease in the willingness to report [16].

Studies examining the impact of transformational leadership on nursing outcomes have primarily addressed this impact through mediators such as structural empowerment, organizational commitment, and job satisfaction; while error reporting and the culture of blame are often identified as problem areas, they have not been modeled as directly measured outcomes [17]. While effective managerial leadership has been associated with increased error reporting by nurses, findings in this area are scattered due to variations in measurement and outcomes, and there is a need for studies grounded in a clear theoretical framework [18]. In this context, studies that address transformational leadership and psychological safety together, while modeling error reporting behavior as a direct outcome, are quite limited, particularly in the emergency department setting.

## 2. Materials and Methods

### 2.1. Purpose of the Study and Hypotheses

**Figure 1:**
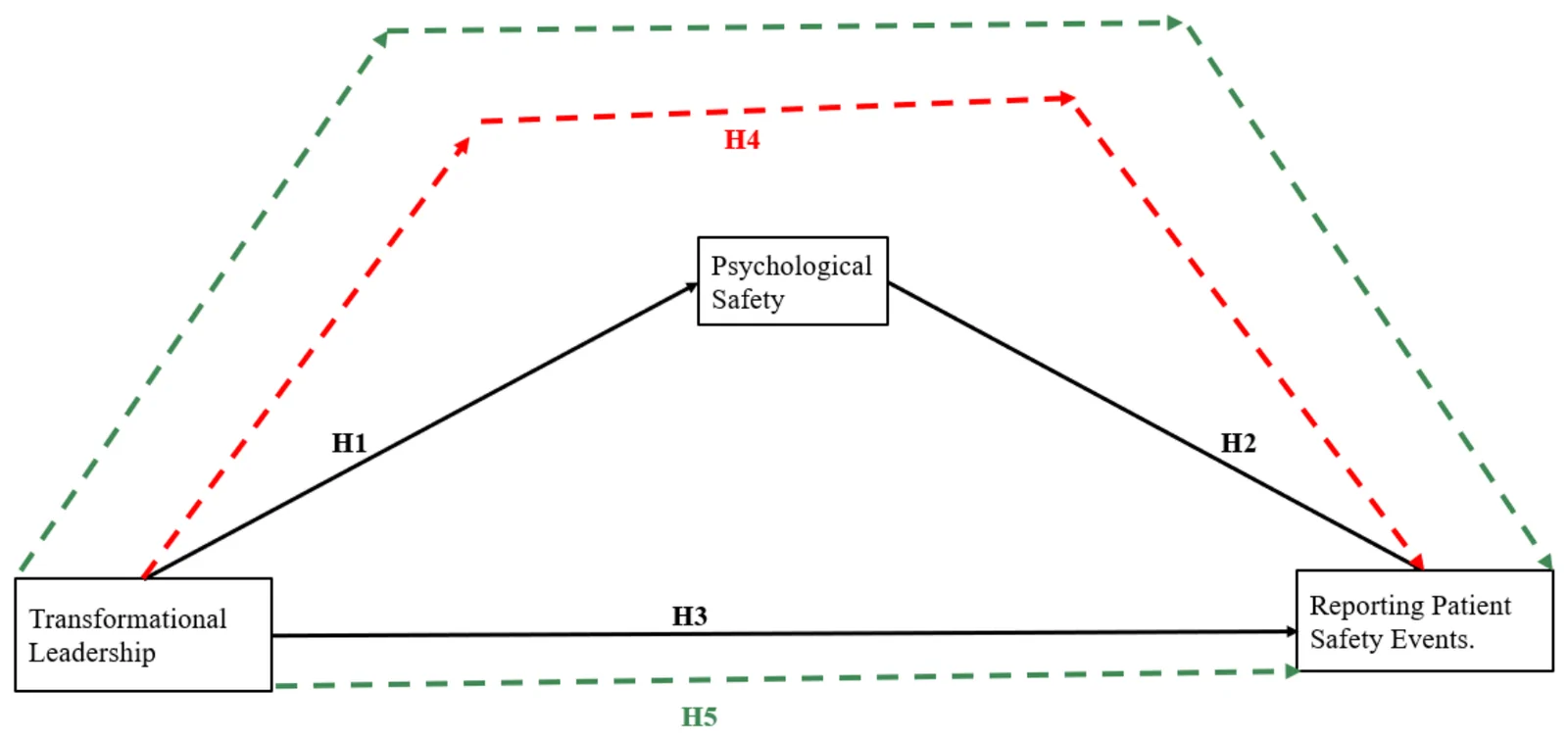
Research model.

The purpose of this study is to examine the relationships among perceived transformational leadership, psychological safety, and patient safety event reporting behavior among emergency department nurses; specifically, to test the mediating role of psychological safety in the relationship between transformational leadership and reporting behavior. Based on the theoretical framework summarized above, the following hypotheses were tested (Figure 1):

**H1.**  *Transformational leadership is positively associated with psychological safety.*

**H2.**  *Psychological safety is positively associated with patient safety event reporting behavior.*

**H3.**  *Transformational leadership is positively associated with patient safety event reporting behavior.*

**H4.**  *Psychological safety mediates the association between transformational leadership and patient safety event reporting behavior.*

### 2.2. Study Population and Sample

This study was conducted in the emergency departments of two city hospitals in Ankara, Turkey. According to information obtained from the head nurses, a total of 450 nurses were employed in these departments at the time of data collection. No sampling procedure was applied; all nurses working in the two emergency departments were invited to participate (total population sampling of the accessible workforce). The inclusion criterion was a minimum of one year of work experience in the emergency department. Data were collected in July 2026. Prior to data collection, the head nurses of the emergency units were consulted and the necessary permissions were obtained. The researchers left 300 data collection forms in the relevant units to be distributed to nurses who met the inclusion criterion and volunteered to participate, and the completed forms were collected after a predetermined period. After the exclusion of five blank or incomplete forms, 295 forms were included in the analysis (completion rate of distributed forms: 98.3%), representing 65.6% of all emergency department nurses. As the number of nurses who did not meet the inclusion criterion or were unavailable during the data-collection period (e.g., on annual leave) could not be determined, the response rate among eligible nurses could not be calculated precisely; however, it was at least 65.6%. Because all accessible nurses were invited to participate, no a priori sample size calculation was performed.

### 2.3. Data Collection Tools

The research data were collected using the “Form for Determining Nurses’ Individual and Professional Characteristics,” developed by the researchers, along with the Psychological Safety Scale, the Transformational Leadership Scale, and the “Reporting Patient Safety Events” composite of the Hospital Survey on Patient Safety Culture 2.0 (HSOPSC 2.0). Weekly working hours were assessed with an open-ended question asking participants to report their average weekly working hours in the emergency department, including on-call shifts. In both hospitals, emergency department nurses worked under a system that included 24-h on-call shifts.

**Transformational Leadership Scale.** The scale developed by Podsakoff et al. [15] was used to measure the level of transformational leadership that participants perceived in their managers. The scale was adapted into Turkish by İşcan [19]. The five-point Likert-type scale consists of 23 items and includes five subscales: vision and inspiration/serving as an appropriate role model, securing acceptance of group goals, intellectual stimulation, showing individual concern, and high-performance expectations. The Cronbach’s α reliability coefficient for the scale as a whole is 0.93; for the subscales, it ranges from 0.82 to 0.93. Because the aim of this study was to examine transformational leadership as an overall leadership style rather than its specific behavioral dimensions, the total scale score was used in the analyses. The use of a single total score was supported by the acceptable fit of a measurement model in which transformational leadership was specified as a second-order factor.

**Psychological Safety Scale.** The 7-item, unidimensional Psychological Safety Scale developed by Edmondson [11] was used to measure nurses’ individually perceived levels of psychological safety. The scale assesses employees’ perceptions of safety regarding interpersonal risk-taking, the ability to express their opinions openly, and the ability to raise concerns in the workplace. The validity and reliability study of the scale in Turkish was conducted by Bülbül, İşiaçık, and Aytaç [20], who reported that the measurement tool is valid and reliable in the Turkish context (Cronbach’s α = 0.87). Participants responded to the items using a five-point Likert-type scale ranging from “Strongly Disagree” (1) to “Strongly Agree” (5). Negatively worded items were reverse-coded prior to scoring, and higher scores indicate greater perceived psychological safety.

**Patient Safety Event Reporting Behavior.** The “Reporting Patient Safety Events” subscale of the Hospital Survey on Patient Safety 2.0 (HSOPSC 2.0), developed by the Agency for Healthcare Research and Quality, was used. The Turkish adaptation, validity, and reliability study of the scale was conducted by Filiz and Yeşildal [10], who reported that its psychometric properties were adequate (subscale Cronbach’s α = 0.74). In both the original and the Turkish versions, this subscale consists of two items assessing how often mistakes that are caught before reaching the patient, or that reach the patient without causing harm, are reported [10]. Items are rated on a five-point scale from 1 (Never) to 5 (Always), and the mean score was used, with higher scores indicating more frequent reporting. The separate item on the number of events reported in the past 12 months is not part of this subscale and was therefore used for descriptive purposes only.

### 2.4. Data Analysis

The data obtained in the study were analyzed using the SPSS 29.0 and AMOS 24.0 statistical software packages. The descriptive characteristics of the nurses were presented using frequency, percentage, mean, and standard deviation values. The distribution of scale scores was evaluated by examining the skewness and kurtosis coefficients, and it was determined that the data met the assumption of normal distribution; accordingly, parametric methods were used in the analyses. The internal consistency of the measurement instruments was tested using Cronbach’s alpha. The relationships between variables were examined using Pearson’s correlation analysis. Before testing the hypothesized relationships, a confirmatory factor analysis (CFA) was conducted in AMOS 24.0 to evaluate the measurement model including all three scales, with transformational leadership specified as a second-order factor comprising its five first-order dimensions. Model fit was evaluated using χ^2^/df (<3)**,** CFI, TLI, IFI, and NFI (≥0.90), and RMSEA (≤0.08) [21]. Convergent validity was assessed using composite reliability (CR ≥ 0.70) and average variance extracted (AVE ≥ 0.50) [22]. Common method bias was assessed using Harman’s single-factor test.

The mediating role of psychological safety in the relationship between transformational leadership and patient safety event reporting behavior was tested using path analysis based on structural equation modeling in the AMOS 24.0 program. The significance of the indirect effect was assessed using the bootstrap method, generated with 5000 resamples, and a 95% confidence interval. The criterion for accepting the indirect effect as statistically significant was that the confidence interval did not include zero. The issue of multicollinearity among the variables was tested using tolerance and VIF values. In addition to the hypothesized model, professional experience and institutional tenure were specified a priori as potential confounders and entered as covariates on both psychological safety and reporting behavior in a sensitivity analysis conducted to evaluate the robustness of the findings.

### 2.5. Ethical Aspects of the Study

Approval for this study was obtained from the Lokman Hekim University Ethics Committee for Non-Interventional Clinical Research (Decision No. 2026/120 and Code No. 2026091). Written permission was obtained from the hospital administrations where the study was conducted. Nurses invited to participate in the study were provided with information about the study, and those who gave their informed consent to participate were included in the study. The nurses’ identifying information was not included in the data collection forms.

## 3. Results

### 3.1. Participant Characteristics

A total of 295 nurses working in the emergency department participated in the study. Of the participants, 177 (60.0%) were female and 118 (40.0%) were male. More than half of the participants were single (55.6%), while 44.4% were married. Most of the participants held a bachelor’s degree (79.7%), followed by a master’s degree (5.1%), health vocational high school education (8.5%), pre-bachelor education (4.4%), and a PhD degree (2.4%). Regarding professional position, 286 participants (96.9%) were clinical/ward nurses, 7 (2.4%) were clinical/responsible nurses, 1 (0.3%) was a nurse manager, and 1 (0.3%) was classified as other. Among the participants, 209 (70.8%) had no children, whereas 86 (29.2%) had children. Most participants reported that they had voluntarily chosen the nursing profession (74.2%), and 25.8% reported otherwise. Regarding work patterns in the emergency department, the majority worked under an on-call/irregular shift system (77.3%), while 15.9% worked rotating shifts, 5.8% worked only during the day, and 1.0% worked only at night. In terms of the areas in which they worked, 35.6% worked in the yellow zone, 25.4% in the red zone, 16.6% in the green zone, 10.2% in triage, 8.8% in the observation/surveillance area, 0.7% in the resuscitation area, and 2.7% reported rotational work. Most participants (76.9%) reported that they had voluntarily chosen to work in the emergency department, whereas 23.1% had not. The mean age of the participants was 29.42 years (SD = 5.89). The mean duration of employment in the current institution was 3.73 years (SD = 2.57), while the mean length of professional experience was 6.28 years (SD = 6.17). The mean reported weekly working hours was 60.75 h (SD = 11.69). Detailed participant characteristics are presented in Table 1.

### 3.2. Descriptive Statistics, Reliability, and Correlations

The descriptive statistics, internal consistency coefficients, and inter-scale correlations are presented in Table 2. The mean scores were 3.17 (SD = 0.84) for transformational leadership, 2.92 (SD = 0.83) for psychological safety, and 2.96 (SD = 0.91) for reporting patient safety events behavior. The skewness and kurtosis values ranged from −0.01 to 0.39 and from −0.50 to −0.25, respectively, indicating no substantial departure from normality. All three scales demonstrated high internal consistency, with Cronbach’s alpha coefficients ranging from 0.89 to 0.96. Transformational leadership was positively and significantly correlated with psychological safety (r = 0.505, *p* < 0.001) and reporting patient safety events behavior (r = 0.383, *p* < 0.001). Psychological safety was also positively correlated with reporting patient safety events behavior (r = 0.511, *p* < 0.001). These findings indicate that higher levels of transformational leadership were associated with greater psychological safety and more frequent reporting of patient safety events behavior, while psychological safety showed a particularly strong positive association with reporting patient safety events behavior. The inter-scale correlations provided preliminary support for examining psychological safety as a potential mediator of the relationship between transformational leadership and reporting patient safety events behavior.

Potential common method bias was assessed before testing the hypothesized structural relationships. Harman’s single-factor test was conducted using an unrotated factor analysis of the study items. The first factor accounted for 40.38% of the total variance, which was below the commonly used 50% criterion. Multiple factors emerged rather than a single dominant factor accounting for most of the variance. These findings did not indicate substantial common method variance according to Harman’s single-factor criterion.

### 3.3. Measurement Model

**Figure 2:**
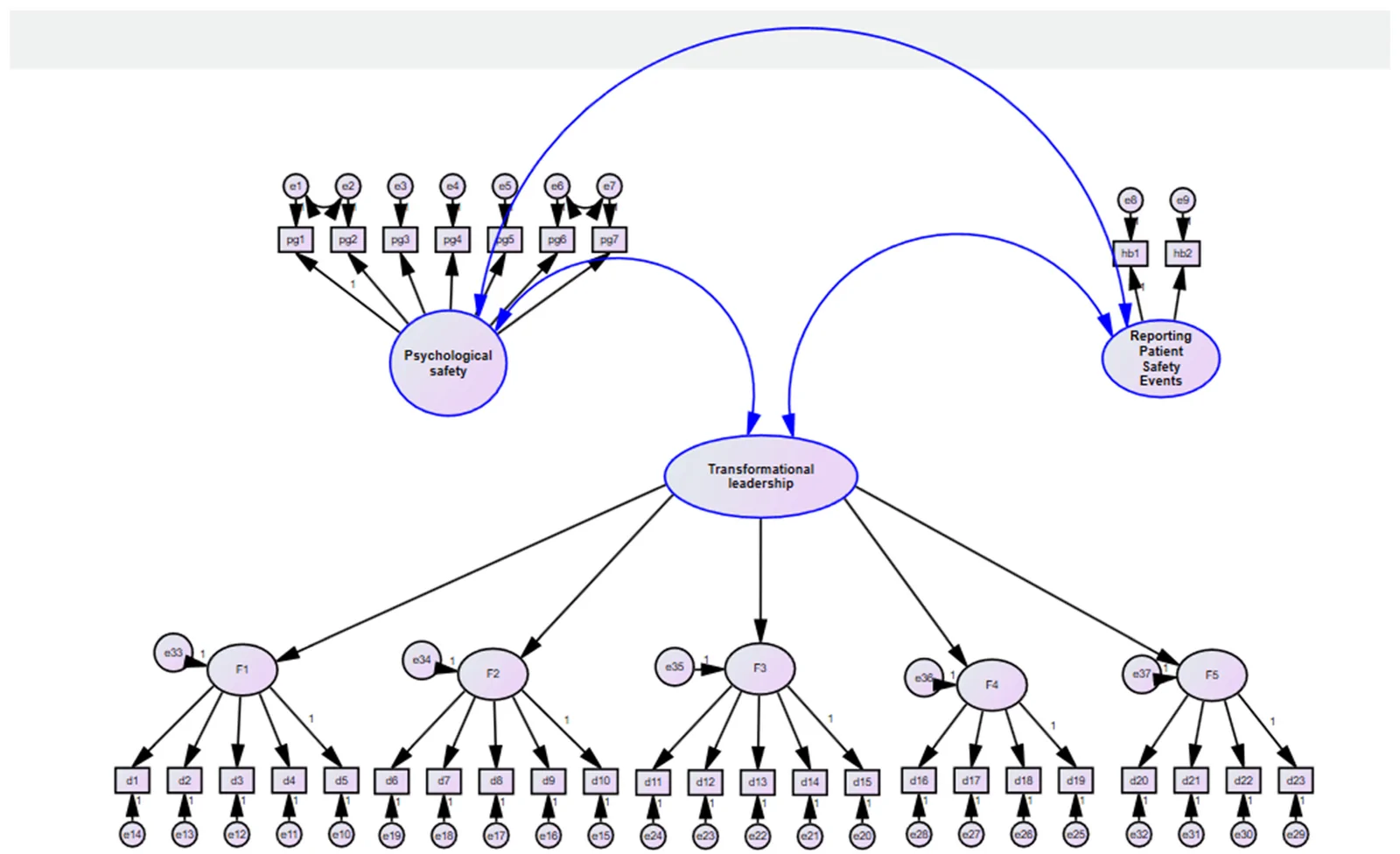
Measurement model of the study scales, with transformational leadership specified as a second-order factor.

The measurement model showed an acceptable fit to the data (χ^2^ = 1248.06, df = 454, χ^2^/df = 2.75, CFI = 0.94, TLI = 0.94, IFI = 0.94, NFI = 0.91, RMSEA = 0.07) (Figure 2; Table 3). All standardized factor loadings ranged from 0.68 to 0.98. CR values ranged from 0.90 to 0.98 and AVE values from 0.70 to 0.89, exceeding the recommended thresholds and supporting convergent validity. Two pairs of error terms within the Psychological Safety Scale were allowed to covary.

### 3.4. Mediation Analysis of Psychological Safety

**Figure 3:**
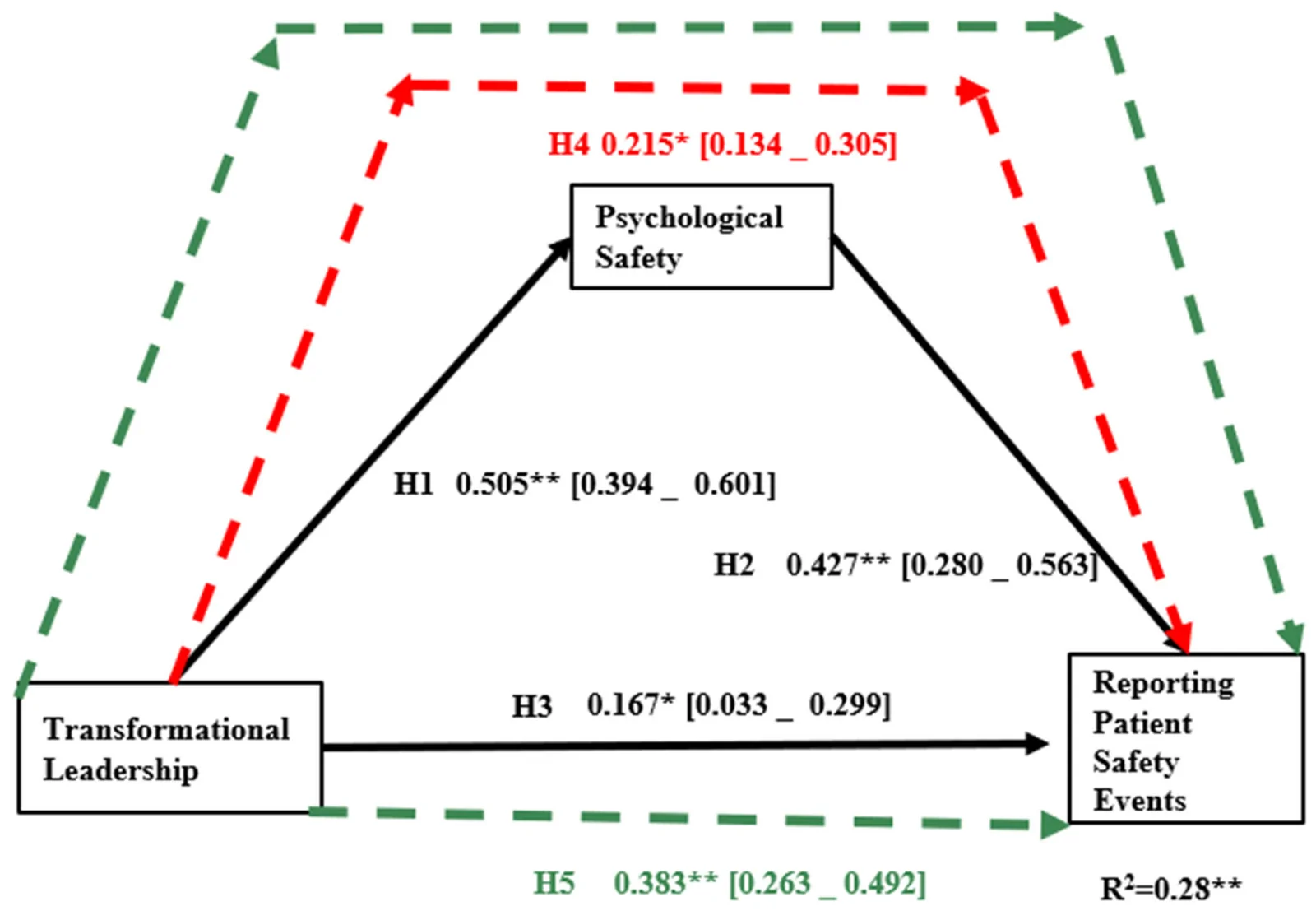
The mediating role of psychological safety in the association between transformational leadership and patient safety event reporting behavior. Note: Solid lines represent direct effects; the red dashed line represents the indirect effect (H4); the green dashed line represents the total effect (H5). Values in brackets are 95% bootstrap confidence intervals. * *p* < 0.05; ** *p* < 0.01.

The hypothesized mediation model was tested in AMOS 24 (Figure 3). The indirect effect was assessed using bootstrapping with 5000 bootstrap samples and a 95% confidence interval. The results indicated that transformational leadership had a significant positive direct effect on psychological safety (β = 0.505, 95% CI [0.394, 0.601], *p* < 0.001), supporting H1 (Table 4). Psychological safety, in turn, had a significant positive direct effect on reporting patient safety events behavior (β = 0.427, 95% CI [0.280, 0.563], *p* < 0.001), supporting H2. Transformational leadership also had a significant positive direct effect on reporting patient safety events behavior (β = 0.167, 95% CI [0.033, 0.299], *p* = 0.016), supporting H3.

The bootstrap analysis demonstrated a significant indirect effect of transformational leadership on reporting patient safety events behavior through psychological safety (β = 0.215, 95% CI [0.134, 0.305]). Because the 95% bootstrap confidence interval did not include zero, the indirect effect was considered statistically significant, supporting H4. The direct effect of transformational leadership on reporting patient safety events behavior remained significant after psychological safety was included in the model (β = 0.167, 95% CI [0.033, 0.299], *p* = 0.016), indicating partial rather than full mediation. The total effect of transformational leadership on reporting patient safety events behavior was also significant (β = 0.383, 95% CI [0.263, 0.492], *p* < 0.001). The model explained 28% of the variance in reporting patient safety events behavior (R^2^ = 0.28). Because the path model included all possible paths among the three observed variables, it was just-identified (df = 0); therefore, model fit was evaluated for the measurement model. Collinearity diagnostics indicated no evidence of problematic multicollinearity among the predictors of reporting patient safety events behavior (transformational leadership and psychological safety)**,** with a tolerance value of 0.745 and a VIF of 1.342. Overall, the findings support the proposed mediation model, indicating that transformational leadership is associated with reporting patient safety events behavior both directly and indirectly through psychological safety (Table 4).

### 3.5. Robustness (Sensitivity) Analysis

As shown in Table 5, the robustness of the findings was assessed in a sensitivity analysis by re-estimating the model with professional experience and institutional tenure entered as covariates on both psychological safety and event reporting. The three focal paths remained significant and virtually unchanged (TL → PS, β = 0.504; PS → RPSE, β = 0.433; TL → RPSE, β = 0.174; all *p* ≤ 0.002), as did the indirect (β = 0.230, 95% CI [0.137, 0.311]) and total (β = 0.383, 95% CI [0.273, 0.504]) effects. Among the covariates, only institutional tenure was associated with reporting, and only weakly (β = −0.048, *p* = 0.019); all other covariate paths were non-significant. These findings indicate that the hypothesized relationships are robust to the inclusion of demographic confounders.

## 4. Discussion

This study was conducted to examine the relationships among transformational leadership, psychological safety, and patient safety event reporting behavior among emergency department nurses. The fact that the nurses participating in the study were young (mean age 29.4 years) and had relatively little professional experience (mean 6.3 years) indicates that the sample consisted primarily of nurses in the early stages of their careers. This finding is also significant in that it suggests that the perception of psychological safety may still be fragile among this group of staff. Indeed, it has been reported that nurses with relatively little clinical experience are less likely to voice their concerns regarding patient safety than experienced nurses [23]. In this study, the mean perceived psychological safety score (2.92) was close to the midpoint of the scale, indicating a moderate level of perceived psychological safety and suggesting room for improvement. Because psychological safety was measured as an individual perception rather than as a shared team climate, this finding reflects nurses’ individual perceptions rather than the collective climate of the emergency departments. Other studies have also reported that nurses’ perceived psychological safety remains at a moderate or below-moderate level [24,25].

The fact that the vast majority of participants (77.3%) worked under an on-call/irregular shift system that included 24-h on-call shifts, combined with the reported high weekly working hours in the emergency department (mean 60.75 h), highlights the unpredictable and exhausting working conditions in the emergency department. These conditions—a combination of irregular work schedules, high workloads, and a relatively inexperienced staff—may create an environment where the likelihood of fatigue-related errors increases [26,27]. Although weekly working hours were not included in the analytical model, their potential role in reporting behavior warrants examination in future studies. In such an environment, the reporting of patient safety events is of critical importance because it enables organizational learning that prevents recurring errors and improves patient safety and the quality of care [28,29]. The most striking finding of this study is the strong relationship between psychological safety and patient safety event reporting behavior (r = 0.511; β = 0.427). This finding suggests that among the variables included in the model, psychological safety showed the strongest association with reporting behavior and that nurses are more likely to report incidents to the extent that they feel psychologically safe. Pfeifer and colleagues [30] also supported this finding by demonstrating that, in pediatrics—a high-risk work environment—psychological safety significantly predicted nurses’ intention to report incidents (β = 0.16). This finding is directly consistent with the theory that positions psychological safety as a fundamental prerequisite for behaviors involving interpersonal risk, such as error reporting [11]. Particularly in the emergency department, where errors can lead to severe consequences—including permanent disability or even death—for patients, nurses’ sense of psychological safety becomes even more critical for error reporting.

The findings of the study indicate that transformational leadership is closely associated with nurses’ perception of psychological safety. The significant and positive direct effect of transformational leadership on psychological safety (β = 0.505) suggests that nurses’ sense of safety may be largely related to the leadership style exhibited by their manager. This finding is also consistent with theoretical expectations: it is argued that the components of transformational leadership—idealized influence, inspirational motivation, intellectual stimulation, and individualized consideration—foster a team climate in which mistakes are not punished and asking questions, expressing concerns, and seeking help are viewed as safe [11,14]. In other words, nurses’ perceived psychological safety may be closely linked to their managers’ behavior. This finding is consistent with the literature. For example, Wang et al. [31] found that transformational leadership is significantly associated with psychological safety. Lee and Dahinten [32] found that inclusive leadership significantly predicted psychological safety (β = 0.40), and that psychological safety, in turn, significantly predicted nurses’ behavior in voicing opinions (β = 0.41) and their intention to report errors (β = 0.19). Asfour et al. [33] demonstrated that servant leadership in critical care is a strong predictor of psychological safety (β = 0.82).

The model revealed that transformational leadership has both a significant direct effect (β = 0.167) on patient safety event reporting behavior and a significant indirect effect (β = 0.215) mediated by psychological safety. Together, these two paths constitute the total effect (β = 0.383), which corresponds to the bivariate correlation between transformational leadership and reporting behavior; the indirect effect thus accounted for approximately 56% of the total effect. The fact that the indirect effect is stronger than the direct effect suggests that psychological safety may be an important mechanism in the relationship between transformational leadership and reporting behavior. However, the fact that the direct effect remains significant indicates that this relationship cannot be explained solely by psychological safety; other mechanisms may also be involved, such as managers explicitly encouraging reporting as an expected behavior. This partial mediation pattern is consistent with findings showing that psychological safety only partially mediates the effect of inclusive leadership on nurses’ voice and error-reporting behaviors [32]. Therefore, while fostering psychological safety may be a central strategy for enhancing reporting in the emergency department, it may not be sufficient on its own. In particular, the direct effect of the leadership style exhibited by the manager should not be overlooked. The transformational leadership–psychological safety–reporting model tested in our study explained 28% of the variation in reporting behavior. However, approximately 72% of the variance remained unexplained, indicating that other factors not included in the model such as organizational safety culture, workload and staffing, managerial support, fear of blame, previous reporting experiences, and institutional reporting systems may also contribute to reporting behavior.

Notably, in the robustness analysis that included confounders, a small but significant negative association was found between length of service and patient safety event reporting behavior (β = −0.048, *p* = 0.019). Although the effect size is limited, this finding suggests that more senior nurses report events less frequently than their less senior counterparts. One possible explanation is burnout, particularly its depersonalization dimension. Prolonged and routine exposure to adverse events in the emergency department may lead to the normalization of these events (normalization of deviance) and the perception that they are not worth reporting. Studies have shown that burnout and emotional exhaustion are negatively associated with incident reporting, at least regarding certain types of reports [34,35]. However, as burnout was not measured in the present study, this explanation remains speculative and should be examined directly in future research.

### Limitations

The findings of this study should be interpreted with certain limitations in mind. The study was conducted using a cross-sectional design, which precludes causal inferences; the findings reflect associations, and longitudinal studies are needed to establish temporal order. Data were collected from the emergency departments of two city hospitals in Ankara. This limits the generalizability of the findings to different types of institutions, regions, and health care systems. Since all variables were assessed using self-report scales completed by nurses, there is a possibility of common method variance and social desirability bias in the data; this tendency may have influenced responses, particularly regarding relatively sensitive topics such as psychological safety and error reporting. Because both the predictor and the outcome were self-reported, common method variance may have inflated the observed associations, so the true effect sizes could be somewhat smaller than those reported; conversely, social desirability may have led to over-reporting of safe behavior, biasing the mean level of the outcome upward without necessarily altering the direction of the modeled relationships. Harman’s single-factor test indicated that a single factor accounted for 40.38% of the total variance, below the 50% threshold; however, this test alone cannot rule out common method variance. In addition, psychological safety was measured as an individual perception rather than as a team-level climate. Finally, the fact that patient safety event reporting behavior was assessed using a two-item indicator limits the measurement scope of this construct, as it captures only the reporting of mistakes that are caught before reaching the patient or cause no harm; thus, the findings may not extend to more serious events. Future studies should use multi-item instruments and objective incident reporting data.

## 5. Conclusions

The reporting of patient safety events is a prerequisite for safe care in the emergency department setting, where the risk of error is high. This study suggests that such reporting is not merely a function of institutional systems or individual responsibility but is also closely associated with nurses’ perceived psychological safety. Psychological safety showed the strongest association with patient safety event reporting behavior among the variables examined (β = 0.427; r = 0.511; *p* < 0.001), and the model explained 28% of the variation in reporting behavior. In other words, nurses appear to be more likely to report events to the extent that they feel safe.

Transformational leadership was also an important factor in this model. The study found that transformational leadership was positively associated with psychological safety (β = 0.505; *p* < 0.001); its association with reporting behavior was both direct and indirect through psychological safety (indirect effect β = 0.215), with the indirect path accounting for more than half of the total association. The fact that a significant portion of the relationship between leadership and reporting behavior was observed through psychological safety (partial mediation) suggests that a safe reporting culture may depend on the relational foundation between leadership and employees. In this regard, the study contributes to the literature as one of the few studies that simultaneously examines the leadership–psychological safety–reporting link within the context of transformational leadership and emergency services.

These findings suggest that strengthening a culture of reporting may require not only establishing a systematic reporting mechanism but also fostering, through management, a climate of trust that encourages nurses to use this mechanism. In this context, the development of transformational leadership skills can be viewed as a promising lever for making patient safety an organizational priority, in addition to factors such as individual attention and education. However, as these implications are derived from cross-sectional associations, longitudinal and intervention studies are needed to determine whether leadership development programs increase reporting in practice.

## Figures and Tables

**Table 1:** Sociodemographic and professional characteristics of the participants.

Gender	n	%	Marital Status	n	%
Male	118	40.0	Married	131	44.4
Female	177	60.0	Single	164	55.6
Educational status	n	%	Job position	n	%
Health vocational high school	25	8.5	Clinical/ward nurse	286	96.9
Pre-bachelor	13	4.4	Clinical/responsible nurse	7	2.4
Bachelor	235	79.7	Nurse manager	1	0.3
Master’s degree	15	5.1	Other	1	0.3
PhD	7	2.4			
Having children	n	%	Chose profession voluntarily	n	%
Yes	86	29.2	Yes	219	74.2
No	209	70.8	No	76	25.8
Working method in the ED	n	%	Area worked in the ED	n	%
Daytime only	17	5.8	Triage	30	10.2
Rotating shifts	47	15.9	Resuscitation	2	0.7
Night shift only	3	1.0	Red zone	75	25.4
On-call/irregular shifts	228	77.3	Yellow zone	105	35.6
Voluntary work in the ED	n	%	Green zone	49	16.6
Yes	227	76.9	Observation/surveillance	26	8.8
No	68	23.1	Rotational work	8	2.7
	Mean	SD		Mean	SD
Age	29.42	5.89	Institutional tenure (years)	3.73	2.57
Professional experience (years)	6.28	6.17	Weekly working hours	60.75	11.69

**Table 2:** Descriptive statistics, internal consistency, and inter-scale correlations.

	Mean	SD	Skewness	Kurtosis	Cronbach’s α	1	2	3
1. Transformational Leadership	3.17	0.84	−0.01	−0.50	0.94	1		
2. Psychological Safety	2.92	0.83	0.16	−0.42	0.96	0.505 **	1	
3. Reporting Patient Safety Events Behavior	2.96	0.91	0.39	−0.25	0.89	0.383 **	0.511 **	1

Note: ** *p* < 0.001. Columns 1–3 report the inter-scale Pearson correlations.

**Table 3:** Confirmatory factor analysis results: composite reliability, average variance extracted, and standardized factor loadings.

Scale and Factor Name	CR	AVE	Loadings of Factor Items
Transformational Leadership F1	0.98	0.89	TL 1: 0.95
			TL 2: 0.94
			TL 3: 0.93
			TL 4: 0.95
			TL 5: 0.95
Transformational Leadership F2	0.97	0.86	TL 6: 0.93
			TL 7: 0.93
			TL 8: 0.93
			TL 9: 0.93
			TL 10: 0.93
Transformational Leadership F3	0.97	0.87	TL 11: 0.95
			TL 12: 0.93
			TL 13: 0.92
			TL 14: 0.93
			TL 15: 0.93
Transformational Leadership F4	0.96	0.84	TL 16: 0.92
			TL 17: 0.91
			TL 18: 0.91
			TL 19: 0.93
Transformational Leadership F5	0.95	0.84	TL 20: 0.90
			TL 21: 0.91
			TL 22: 0.92
			TL 23: 0.93
Psychological Safety	0.94	0.70	PS 1: 0.68
			PS 2: 0.76
			PS 3: 0.93
			PS 4: 0.98
			PS 5: 0.96
			PS 6: 0.79
			PS 7: 0.72
Reporting Patient Safety Events	0.90	0.81	RPSE 1: 0.92
			RPSE 2: 0.88

Goodness-of-fit indices: χ^2^ = 1248.06; df = 454; χ^2^/df = 2.75; CFI = 0.94; TLI = 0.94; NFI = 0.91; IFI = 0.94; RMSEA = 0.07.

**Table 4:** Mediation analysis of psychological safety in the relationship between transformational leadership and reporting patient safety events.

Hypothesis	Path	Std. β	CI Lower	CI Upper	Tolerance	VIF	*p*	Decision
H1	TL → PS (direct)	0.505	0.394	0.601			<0.001	Accepted
H2	PS → RPSE (direct)	0.427	0.280	0.563	0.745	1.342	<0.001	Accepted
H3	TL → RPSE (direct)	0.167	0.033	0.299	0.745	1.342	0.016	Accepted
H4	TL → PS → RPSE (indirect)	0.215	0.134	0.305			<0.001	Accepted
-	TL → RPSE (total)	0.383	0.263	0.492			<0.001	-

Note: 95% CI reported as lower and upper bounds. R^2^ = 0.28. TL = Transformational Leadership; PS = Psychological Safety; RPSE = Reporting Patient Safety Events.

**Table 5:** Inclusion of confounding variables in the model.

Path	β (Estimate)	C.R.	*p*	Outcome
TL → PS	0.504	9.89	<0.001	Significant ***
PS → RPSE	0.433	7.64	<0.001	Significant ***
TL → RPSE	0.174	3.04	0.002	Significant **
Professional experience → PS	0.001	0.13	0.894	Non-significant
Institutional tenure → PS	0.013	0.66	0.511	Non-significant
Professional experience → RPSE	0.006	0.72	0.473	Non-significant
Institutional tenure → RPSE	−0.048	−2.34	0.019	Small negative, significant *

Note: * *p* < 0.05; ** *p* < 0.01; *** *p* < 0.001. TL = Transformational Leadership; PS = Psychological Safety; RPSE = Reporting Patient Safety Events.

## Data Availability

The data presented in this study are available on request from the corresponding author. The data are not publicly available due to privacy restrictions.

## References

[1] Asplin B.R., Magid D.J., Rhodes K.V., Solberg L.I., Lurie N., Camargo C.A. (2003). A conceptual model of emergency department crowding. Ann. Emerg. Med..

[2] Nafe P., Habibi-Soola A., Aghaei M.H., Mehri S. (2025). The relationship between error experience and patient safety culture with safe activities of emergency nurses. Sci. Rep..

[3] Khaleghi P., Akbari H., Masoudi Alavi N., Motalebi Kashani M., Batooli Z. (2022). Identification and analysis of human errors in emergency department nurses using SHERPA method. Int. Emerg. Nurs..

[4] Monfaredi S., Gaeeni M., Koohpaei A., Khandan M. (2021). Identification and assessment of nursing task errors in emergency department using SHERPA technique and offering remedial strategies. Int. Emerg. Nurs..

[5] Reason J. (2000). Human error: Models and management. BMJ.

[6] Munn L.T., Lynn M.R., Knafl G.J., Willis T.S., Jones C.B. (2023). A study of error reporting by nurses: The significant impact of nursing team dynamics. J. Res. Nurs..

[7] Brabcová I., Hajduchová H., Tóthová V., Chloubová I., Červený M., Prokešová R., Malý J., Vlček J., Doseděl M., Malá-Ládová K. (2023). Reasons for medication administration errors, barriers to reporting them and the number of reported medication administration errors from the perspective of nurses: A cross-sectional survey. Nurse Educ. Pract..

[8] Hamed M.M.M., Konstantinidis S. (2022). Barriers to Incident Reporting among Nurses: A Qualitative Systematic Review. West J. Nurs. Res..

[9] Rutledge D.N., Retrosi T., Ostrowski G. (2018). Barriers to medication error reporting among hospital nurses. J. Clin. Nurs..

[10] Filiz E., Yeşildal M. (2022). Turkish adaptation and validation of revised Hospital Survey on Patient Safety Culture (TR–HSOPSC 2.0). BMC Nurs..

[11] Edmondson A. (1999). Psychological safety and learning behavior in work teams. Adm. Sci. Q..

[12] Edmondson A.C. (1996). Learning from mistakes is easier said than done: Group and organizational influences on the detection and correction of human error. J. Appl. Behav. Sci..

[13] Bass B.M., Riggio R.E. (2006). Transformational Leadership.

[14] Bass B.M. (1985). Leadership and Performance Beyond Expectations.

[15] Podsakoff P.M., MacKenzie S.B., Moorman R.H., Fetter R. (1990). Transformational leader behaviors and their effects on followers’ trust in leader, satisfaction, and organizational citizenship behaviors. Leadersh. Q..

[16] Dirik H.F., Theiakouli C., Efstathiou N. (2026). The Influence of Nursing Leadership Styles on Medication Safety: A Mixed-Methods Systematic Review. Int. Nurs. Rev..

[17] Ystaas L.M.K., Nikitara M., Ghobrial S., Latzourakis E., Polychronis G., Constantinou C.S. (2023). The Impact of Transformational Leadership in the Nursing Work Environment and Patients’ Outcomes: A Systematic Review. Nurs. Rep..

[18] Lee S.E., Hyunjie L., Sang S. (2023). Nurse Managers’ Leadership, Patient Safety, and Quality of Care: A Systematic Review. West J. Nurs. Res..

[19] İşcan Ö.F. (2002). Küresel Işletmecilikte Dönüştürücü Liderlik Anlayışı: Büyük Ölçekli Işletmelerde Bir Uygulama [Yayımlanmamış Doktora Tezi].

[20] Bülbül S., İşiaçık S., Aytaç S. (2022). Measurement of perceived psychological safety: Integration, review and evidences for the scale in the context of Turkey. J. Econ. Cult. Soc..

[21] Hu L.T., Bentler P.M. (1999). Cutoff criteria for fit indexes in covariance structure analysis: Conventional criteria versus new alternatives. Struct. Equ. Model. Multidiscip. J..

[22] Fornell C., Larcker D.F. (1981). Evaluating structural equation models with unobservable variables and measurement error. J. Mark. Res..

[23] Lee E., Kwon H. (2025). Impact of speaking-up climate on Korean nurses’ voice and silence: Focusing on nurses’ clinical career stages. Collegian.

[24] Kamaluddin K.G. (2025). Psychological Safety, Level of Burnout, and Intention to Stay among Staff Nurses in a Tertiary Hospital in the Kingdom of Saudi Arabia. J. Nurs. Sci. Prof. Pract..

[25] Tai C., Chen D., Zhang Y., Teng Y., Li X., Ma C. (2024). Exploring the influencing factors of patient safety competency of clinical nurses: A cross-sectional study based on latent profile analysis. BMC Nurs..

[26] Farag A., Gallagher J., Carr L. (2024). Examining the Relationship Between Nurse Fatigue, Alertness, and Medication Errors. West. J. Nurs. Res..

[27] Son Y.-J., Lee E.K., Ko Y. (2019). Association of Working Hours and Patient Safety Competencies with Adverse Nurse Outcomes: A Cross-Sectional Study. Int. J. Environ. Res. Public Health.

[28] Archer S., Hull L., Soukup T., Mayer E., Athanasiou T., Sevdalis N., Darzi A. (2017). Development of a theoretical framework of factors affecting patient safety incident reporting: A theoretical review of the literature. BMJ Open.

[29] Kumah A., Zon J., Obot E., Yaw T.K., Nketsiah E., Bobie S.A. (2024). Using Incident Reporting Systems to Improve Patient Safety and Quality of Care. Glob. J. Qual. Saf. Healthc..

[30] Pfeifer L., Vessey J., Cazzell M., Ponte P.R., Geyer D. (2023). Relationships among psychological safety, the principles of high reliability, and safety reporting intentions in pediatric nursing. J. Pediatr. Nurs..

[31] Wang H.F., Chen Y.-C., Yang F.-H., Juan C.-W. (2021). Relationship between transformational leadership and nurses’ job performance: The mediating effect of psychological safety. Soc. Behav. Personal. An. Int. J..

[32] Lee S.E., Dahinten V.S. (2021). Psychological Safety as a Mediator of the Relationship Between Inclusive Leadership and Nurse Voice Behaviors and Error Reporting. J. Nurs. Scholarsh..

[33] Asfour L., Saleh A.M.d., Abu-Wardeh Y. (2025). The Role of Servant Leadership in Fostering Psychological Safety Among Nurses in Jordanian Hospitals. J. Nurs. Manag..

[34] Halbesleben J.R.B., Wakefield B.J., Wakefield D.S., Cooper L.B. (2008). Nurse Burnout and Patient Safety Outcomes: Nurse Safety Perception Versus Reporting Behavior. West. J. Nurs. Res..

[35] Poku C.A., Ahiadzese Y.A., Oyortey H.K., Lamptey S.N.O., Asante J., Lumor D.A., Hagan D. (2026). Effects of Toxic Leadership Behavior and Emotional Exhaustion on Patient Safety Incident Reporting. J. Nurs. Manag..

